# Coverage of diarrhoea-associated *Escherichia coli* isolates from different origins with two types of phage cocktails

**DOI:** 10.1111/1751-7915.12113

**Published:** 2014-02-14

**Authors:** Gilles Bourdin, Armando Navarro, Shafiqul A Sarker, Anne-C Pittet, Firdausi Qadri, Shamima Sultana, Alejandro Cravioto, Kaisar A Talukder, Gloria Reuteler, Harald Brüssow

**Affiliations:** 1 Nutrition and Health Department, Nestlé Research CenterLausanne, Switzerland; 2 Department of Public Health, Faculty of Medicine, Universidad Nacional Autónoma de MéxicoMexico City, Mexico, Mexico; 3 International Center for Diarrheal Disease ResearchBangladesh, Dhaka, Bangladesh

## Abstract

Eighty-nine T4-like phages from our phage collection were tested against four collections of childhood diarrhoea-associated *Escherichia coli* isolates representing different geographical origins (Mexico *versus* Bangladesh), serotypes (69 O, 27 H serotypes), pathotypes (ETEC, EPEC, EIEC, EAEC, VTEC, *Shigella*), epidemiological settings (community and hospitalized diarrhoea) and years of isolation. With a cocktail consisting of 3 to 14 T4-like phages, we achieved 54% to 69% coverage against predominantly EPEC isolates from Mexico, 30% to 53% against mostly ETEC isolates from a prospective survey in Bangladesh, 24% to 61% against a mixture of pathotypes isolated from hospitalized children in Bangladesh, and 60% coverage against *Shigella* isolates. In comparison a commercial Russian phage cocktail containing a complex mixture of many different genera of coliphages showed 19%, 33%, 50% and 90% coverage, respectively, against the four above-mentioned collections. Few O serotype-specific phages and no broad-host range phages were detected in our T4-like phage collection. Interference phenomena between the phage isolates were observed when constituting larger phage cocktails. Since the coverage of a given T4-like phage cocktail differed with geographical area and epidemiological setting, a phage composition adapted to a local situation is needed for phage therapy approaches against *E. coli* pathogens.

## Introduction

The antibiotic crisis has rekindled the interest in phage therapy approaches that were developed in Eastern Europe (Brüssow, 2012). Phage therapy has theoretical advantages over antibiotic therapy. Phage is a self-amplifiable antibacterial agent that increases in titer only when it meets *in vivo* its target (Payne *et al*., 2000). Phage is quickly eliminated from the body in the absence of the pathogen as shown in pioneer work in mice (Merril *et al*., 1996) and later in humans (Bruttin and Brüssow, 2005). In addition, phages are mostly species-specific bacterial viruses. Exceptions are phages infecting *Listeria innocua* and *Listeria monocytogenes* or *Salmonella* and *Escherichia coli* (Santos *et al*., 2010). Consequently, and in notable contrast to antibiotics, no collateral damage on the commensal microbiota is to be expected. However, the full scale of ecological relationships between coexisting bacterial species in a given microbial community is quite complex and is not fully described or understood yet. Data from human healthy subjects do not suggest phage-induced gut microbiota changes induced by oral phage (Sarker *et al*., 2012; McCallin *et al*., 2013). The specificity of phages has also a downside: many phages are not only species-specific, but are also strain-specific. This property becomes a coverage problem when phage therapy targets a disease caused by a complex pathogen like diarrhoea-associated *E. coli*, which is represented by many different pathotypes and serotypes (Chibani-Chennoufi *et al*., 2004a,b). The constitution of complex phage cocktails then becomes also a commercial problem of producing a standardized preparation. For some bacterial pathogens, broad host-range phages have been described (*Salmonella*: Santos *et al*., 2010; *Staphylococcus aureus*: Kelly *et al*., 2011). However, no such broad host-range phages have so far been isolated for *E. coli*. Recent data suggest that coliphage phi92, isolated from an encapsulated K1 *E. coli* strain and belonging to the novel genus of rv5-like phages, might have a broad host range (Schwarzer *et al*., 2012) like the multivalent *Salmonella* phage PVP-SE1 infecting both *Salmonella* and *E. coli* (Santos *et al*., 2010). These phages have not yet been tested against large collections of pathogenic *E. coli* strains. Phage therapy against *E. coli* must therefore rely on phage cocktails. The structure of host–phage interaction matrices has recently become an active research area (Flores *et al*., 2011; Weitz *et al*., 2013). Different types of interaction were identified: specialist phages that may infect a unique host leading to a nearly diagonal matrix when columns indicate phage and rows host isolates; phage–host interactions that may describe block-like matrices, which exhibit high degrees of modularity; generalist phages and phage–host interaction that are random.

For regulatory reasons, we worked with a cocktail containing only a single phage genus, reasoning that it might be easier to demonstrate safety for a single phage group than for a cocktail containing numerous different phage genera. We settled for the T4-like genus of *Myoviridae* (Bruttin and Brüssow, 2005; Sarker *et al*., 2012) because professional virulent phages that destroy the host genome during the infection process are considered by many researchers as best candidates for phage therapy (Brüssow, 2012). Early on, it was reported that T4-like phages recognize lipopolysaccharides (LPS) in the outer membrane of *E. coli* (O antigens) as primary phage receptors (Tamaki *et al*., 1971). However, LPS synthesizing genes represent a hotspot of genetic diversity in *E. coli* (Heinrichs *et al*., 1998). In fact, the specificity of LPS became the basis for the definition of more than 150 different O serotypes, although not all O serotypes are encountered in pathogenic *E. coli* (Robins-Browne, 1987). If LPS is the receptor for T4-like phages on *E. coli*, one would predict problems for achieving high coverage of a phage cocktail against diarrhoea-associated *E. coli*. Other researchers identified the outer membrane protein OmpC as receptor (Hashemolhosseini *et al*., 1994; Yu *et al*., 2000) for T4 phages. Since this gene is well conserved across different genera of *Enterobacteriaceae* when using the *E. coli* K-12 gene in BLAST searches, multivalent T4-like phages should be expected. In the present report, we explored the coverage of phage cocktails composed of T4-like phages against *E. coli* isolates from different epidemiological settings compared with a commercial Russian phage cocktail containing many different genera of coliphages (McCallin *et al*., 2013). We conclude that pathogen coverage is dependent on the epidemiological context requiring flexible and locally adapted phage cocktails.

## Materials and methods

### Phages

Our T4-like phage collection was described in Sarker and colleagues (2012), and the commercial Russian ColiProteus phage cocktail from Microgen (product number 460502100204; batch numbers 16/ 0905/09. 06 and 3/0307/03. 09) was described in McCallin and colleagues (2013). ColiProteus contains phages directed against *Proteus* and *E. coli*. Sextaphage was another commercial phage cocktail from Microgen (product number 4602784002612 batch numbers 531/ 0211 /III 13 and 196–17 /1205/I 08). Sextaphage (also called Pyobacteriophage Polyvalent by Microgen) contains phages directed against six pathogens: *Staphylococcus aureus*, *Streptococcus pyogenes*, *Proteus mirabilis* and *Proteus vulgaris*, *Pseudomonas aeruginosa*, *Klebsiella pneumoniae*, and *E. coli*.

### Phage propagation and lysis tests

The test phages were propagated on the *E. coli* strain K-12 derivative K803, which lacks prophage lambda, obtained from E. Kutter, Evergreen College, Olympia, Wa, USA. Luria-Bertani (LB) broth was inoculated with 1% of an overnight bacterial culture and incubated at 37°C for 2 h to reach 1 × 10^8^ cfu/ml. Phage lysate was added to the culture to get 1 to 5 × 10^8^ pfu/ml and the infected culture was incubated at 37°C for 3 to 5 further hours with agitation (100 r.p.m.). After removal of bacterial cells and cellular debris by centrifugation for 15 min at 14 000 × g and passing the lysate through a 0.22 μm Millipore filter, the phage titers in the lysates were determined by the plaque assay as described previously (Bruttin and Brüssow, 2005). In small experimental series 100 μl of an appropriate phage dilution and 100 μl of an exponentially growing test pathogenic *E. coli* culture to achieve an moi of 3 were incubated at 37°C in 10 ml tubes containing LB broth, and the OD development was read at different time points against the uninfected test cell as control. For larger experimental series (100 phage isolates against about 100 *E. coli* isolates at three moi in duplicates resulting in 60 000 tests) we used the microtiter lysis test; 96-well plates with 10 μl of centrifugation-cleared and sterile-filtered (0.22 μm pore size) phage lysate and 180 μl of fresh LB broth were inoculated with 20 μl of the test *E. coli* target strains to obtain an moi of 0.1, 0.01 and 0.001 respectively. The plate was incubated at 37°C for 4 h under agitation (200 r.p.m.). Every hour the OD_595_ was measured in a Dynex reader (MRX model, Magellan Biosciences, Chantilly, VA). The *E. coli* strain without phage and non-inoculated LB medium were used as positive and negative controls respectively.

### *E. coli* strains from Mexico

Several hundred *E. coli* test strains were collected between 1985 and 2003 from children who experienced an episode of diarrhoea in two regions of Mexico (Mexico City and the State of Morelos). Eighty-nine strains representing the diversity of O and H serotypes encountered were selected for phage susceptibility testing. The *E. coli* strains isolated from children in the State of Morelos belonged to a field study reported previously (Cravioto *et al*., 1990). In short, the isolates were characterized as *E. coli* strains by the Vitek automated bacterial identification system from BioMérieux. The strains were then serotyped by agglutination assays (Ørskov and Ørskov, 1984) using 96-well microtitre plates and rabbit serum (SERUNAM) obtained against 187 somatic antigens and 53 flagellar antigens for *E*. *coli*, as well as against 45 somatic antigens for *Shigella* species. Polymerase chain reaction (PCR) assays were used to determine the presence of virulence genes with primers specified in Supplementary Table 1. The presence of the following genes was investigated: for enteropathogenic *E*. *coli* (EPEC), intimin gene (*eae*) (Karch *et al*., 1993) and bundle forming pilus (*bfp*) gene (Gunzburg *et al*., 1995); for enterotoxigenic *E. coli* (ETEC), heat-labile enterotoxin LTh (Schultsz *et al*., 1994), heat-stable enterotoxins STh and STp genes (Schultsz *et al*., 1994); and for enterohemorrhagic *E. coli* (EHEC), the Shiga-like toxin Stx genes (Pollard *et al*., 1990). The bacterial strains were stored at −20°C on ‘Protect Bacterial Preservers’ beads (Technical Service Consultants, Lancashire, UK).

**Figure 1:**
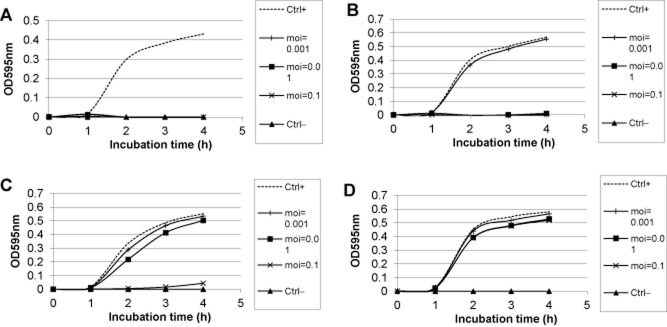
OD development in microtiter wells inoculated with a given pathogenic *E. coli* strain and given a T4-like phage added at three different multiplicities of infection (moi: 0.001, 0.01 and 0.1) in comparison with an uninoculated well (Ctrl−) and uninfected control culture (Ctrl+).A. The phage got a lysis score of three because it lyses at all three moi.B. The phage got a lysis score of two because it lyses only at the two higher moi.C. The phage got a lysis score of one because it lyses only at the highest moi.D. It showed a bacterium-phage pair where no lysis was observed at any of the three moi.

**Table 1:** Host-range determination of different T4-like coliphage cocktails and a commercial Russian phage cocktail on a collection of *E. coli* strains isolated from children with diarrhoea in Mexico

O:H type	VF	N 1-p	N 3-p	N 6-p	N 9-p	Micro	N 10-p
O1:H45	*eae1*				2		
O1:H45	*eae1*	1	1	1	3		1
O2:H4	*eae1*	3					
O4:H16	*eae1*				1	1	
O5:H11	*bfp*	3	3	2	3	1	2
O6:H16	STh, LTh	3	3	1	2		2
O6:H-	*eae1*		2	3	3	1	1
O8:H9	STh, LTh	3					
O8:H-	STh, LTh	3					
O8:H11	nt	2					
O9:H25	nt	1					
O11:H27	*eae1*		2	3	3		
O11:H-	*eae1*		2	3	3		
O11:H-	nt	3	1	2	2		
O11:H?	nt	3		2	1		1
O11:H10	nt	3		3	3		1
O15:H18	*eae1*				1		
O15:H1	nt	2	1	3	3		1
O15:H32	nt	3	1	2	3		
O16:H?	*eae1*		1	1	1		2
O18ac:H-	*eae1*	3	1	1		3	1
O20:H13	*eae1*			1	1		
O22:H1	nt			1	3		1
O23:H15	*eae1*	3	2	3	3		
O25:H-	*eae1*		1	3	3		
O25:H4	nt	3	3	3	3	3	1
O26:H11	*eae1*		3	3	2		1
O26:H8	nt			1			
O27:H20	STp	1	3	3	3		3
O28ac	*bfp*	3	1	2	1		
O29:H21	*eae1*	3	1	1			
O38:H9	*eae1*	3	3	3	3		3
O39:H12	*eae1*			2	1		
O45:H-	*eae1*	2	3	3	2		1
O48:H30	nt		1	1	1		
O55:H6	*eae1*	3					
O63:H-	*eae1,bfp*	1	3	3	3		
O75:H5	*eae1*	1	1	3	3	1	1
O78:H10	STh, LTh	3	3	3	3		
O78:H10	STh, LTh		3	3	3		1
O80:H26	STp, LTh,*eae1*	1					
O82:H8	*eae1*						
O84:H?	*eae1*	1					
O85:H10	*eae1*	3	3	3	3	3	2
O86:H18	*eae1*	3	3	3	3	3	2
O86:H18	*eae1*						
O88:H10	*eae1*		3	3	3		2
O100:H25	STh, LTh						
O101:H-	*eae1*					1	1
O103:H43	*eae1*		1	2	3		2
O103:H-	STh, LTh	1					1
O104:H-	*eae1*		3	3	2		
O105ab:H5	*bfp*		3	3	3		
O110:H16	*eae1*		2	2	3		3
O111ab:H-	*eae1*		2	3	3		
O112ac:H9	*eae1*			1			
O113:H21	LTh		3	3	3		3
O113:H-	*bfp*			1			
O113:H-	*eae1, bfp*	3					
O114:H49	nt		1	3	3		3
O117:H27	SLT	1	1	2	2	2	1
O118:H-	*bfp*	3			1	1	
O119:H4	nt		3	3	3		3
O120:H27	*eae1*		1	2	1		
O121:H19	*eae1*						
O125ab:H33	*eae1*						
O125ab:H18	nt			1	3	3	2
O125ac:H-	nt			1			
O126:H21	nt		2	2	1		
O127:H11	*eae1*	3	3	3	3	2	1
O128:H27	*eae1*		3	3	3		3
O132:H8	*bfp*	1	3	3	3		2
O135:H?	*bfp*						3
O141:H32	*eae1*			1	1		1
O141:H26	nt	3	1	2	2		
O142:H?	*eae1*						
O145:H45	*eae1*						
O146:H8	*eae1*		2	3	3		3
O147:H32	nt	3	3	3	3		3
O148:H28	STp, STh, LTh	3	3	3	3	1	3
O149:H-	*eae1*			3	2		
O153:H12	*eae1*					1	
O158:H9	*eae1*			2	1	1	
O159:H25	STp,LTh	2	3	3	3		
O163:H19	*eae1*	1		3	3		
O165:H18	*eae1*	1	3	3	3		3
O166:H5	*eae1, bfp*	3				1	
O167:H5	LTh	3					
O169:H-	STp	3					2

Notes: All isolates were *E. coli* strains identified by the Vitek automated bacterial Identification system (BioMerieux) O:H: The O and H serotypes were identified by reference sera VF (virulence factors): The presence of the following virulence genes from diarrhoea-associated bacteria were assessed by PCR: enterotoxigenic *E. coli* (ETEC) were diagnosed by the presence of the heat-labile enterotoxin LTh (encoded by the *eltA/B* genes) or heat-stable enterotoxins STh (encoded *estA* gene) or STp (encoded by the *st1* gene); enteropathogenic E. coli (EPEC) were diagnosed by the presence of the intimin (encoded by the *eae 1* gene) and the bundle forming pilus (*bfp*) gene; the strains were also tested for the presence of the gene encoding the Shiga-like toxin (SLT); nt: not tested N1-p to N10-p: The indicated *E. coli* strains were tested for lysis graded by 1, 2 and 3 (according to the OD decrease observed at different moi, see Fig. 1) by the following phage cocktails: N1-phage (NPC1001), N3-phage (NPC1001, NPC 1005, NPC1006), N6-phage (NPC1001, NPC 1005, NPC 1006, NPC 1002, NPC 1008, NPC 1003), N9-phage (NPC 1000, NPC 1002, NPC 1003, NPC 1006, NPC 1007, NPC 1008, NPC 1009, NPC 1024, NPC 1031), N10-phage cocktail (NPC 1000, NPC 1001, NPC 1002, NPC 1003, NPC 1004, NPC 1005, NPC 1006, NPC 1007, NPC 1008, NPC 1009) (for the phage codes see Sarker *et al*., 2012) and the commercial Microgen ColiProteus phage cocktail Micro (containing an unknown number of phage isolates representing according to metagenomic analysis at least ten different phage genera, representing all three major families of tailed phages, McCallin *et al*., 2013).

### *E. coli* strains from Bangladesh

A prospective community-based study was conducted in an urban slum in Mirpur, Dhaka, Bangladesh, from April 2002 to October 2004. Pregnant mothers were screened and 300 newborn children were followed prospectively for diarrhoeal diseases (Qadri *et al*., 2007). *E. coli* colonies were isolated as described previously (Albert *et al*., 1995; Qadri *et al*., 2000). In case of diarrhoea, six lactose-fermenting *E. coli* colonies from MacConkey agar were inoculated on GM1-coated microtiter plates containing LB broth for 18 h. The supernatant was tested for ST using an inhibition ELISA procedure (Svennerholm *et al*., 1986) and for LT by using an anti-LT monoclonal antibody (Svennerholm and Wiklund, 1983). ETEC strains were cultured on CF antigen agar (McConnell *et al*., 1989) and tested for the expression of CFA/I, CS1 to CS8, CS12, CS14, CS17, CS19, and CS21 by monoclonal antibody-based dot blot assay (Qadri *et al*., 2000).

## Results

### *E. coli* pathogens from a community survey of childhood diarrhoea in Mexico

Since LPS, the chemical determinant of the somatic O antigen, was identified as receptor for T4-like phages on *E. coli* cells by some authors (Tamaki *et al*., 1971; Thomassen *et al*., 2003), we first tested our T4-like phage collection against a collection of O serotype-defined, diarrhoea-associated *E. coli* strains. *E. coli* strains were isolated from children with diarrhoea living in two regions of Mexico (Cravioto *et al*., 1990). From this collection, we chose 89 *E. coli* strains for screening against our T4-like phage collection (Table 1). The test *E. coli* strains represented 69 different O serotypes. Six O serotypes were associated with two or three different H serotypes. Overall, 26 different H serotypes were observed, several H serotypes were associated with up to 4 O serotypes (the strains were selected for maximal serotype diversity). Fifty isolates showed the EPEC-specific *eae1* gene. Only three of them also showed the *bfp* gene characterizing them as classical EPEC strains, making the majority of the Mexican isolates atypical EPEC. Fourteen isolates showed enterotoxin genes – the most prevalent combination were strains with STh+LTh genes (*n* = 7). Only few strains showed STp+LTh gene combination (*n* = 2), only LTh (*n* = 2), only STp (*n* = 2) and STp+STh+LTh genes (*n* = 1). Compared to the 50 EPEC isolates, only 14 isolates were thus diagnosed as ETEC strains. Shiga-like toxin was found in a single isolate.

### Coverage of the Mexican *E. coli* pathogens with coliphages

The susceptibility of the individual 89 *E. coli* strains for the 98 T4-like phages from our collection (Sarker *et al*., 2012) was tested in a lysis test at three different multiplicities of infection (moi = 0.1, 0.01, 0.001). The lytic effect of the phage was rated according to the observed lysis: grades 1, 2 and 3 lysis represented growth inhibition only at the highest moi, at two and all three moi levels, respectively (Fig. 1). Most T4-like phages had only a narrow host range on the 89 serotyped test strains, but no clear correlation with O serotypes was seen (data not shown). A few phage isolates lysed many *E. coli* strains independent of the O serotype (Supplementary Table 2). The T4-like phage with the broadest host range showed lytic activity on 44 bacterial strains (49% of the total). When combining three T4-like phages with the broadest individual host ranges, an arithmetical coverage of 87% should have been achieved. However, the physical combination of these three T4-like phages only resulted in the lysis of 54% of the *E. coli* strains suggesting interference between the phages (Table 1). When six T4-like phages were combined into a cocktail, we could increase the coverage to 69%. Further increasing the number to nine phages did not result in enlarged coverage (Table 1). A commercial Russian phage cocktail (Coli-Proteus phage cocktail from Microgen) only lysed 17 of the 89 test bacteria (19% coverage) (Table 1).

**Table 2:** Lysis of pathogenic *E. coli* strains isolated from children who experienced a bout of diarrhoea in the community, which did not lead to hospitalization (prospective study from Dhaka/Bangladesh)

No	Toxins	CF	O type	N 9-p	N 14-p	Microgen
1	LT+ST+	CS2+CS3	O6		L	
2	LT+ST+	CS1+CS3,CS21	O6	L	L	
3	LT+ST+	CS2+CS3	O6	L	L	
4	LT+ST+	CS5+CS6			L	
5	LT+ST+	CS1+CS3,CS21	O6	L	L	
6	LT+ST+	CS14				
7	LT+	CS17				L
8	LT+ST+	CS2+CS3	O6		L	
9	ST+	CFA/I	O126			
10	ST+	CS5+CS6	O115			L
11	ST+	CFA/I	O126		L	L
12	LT+ST+	CS4+CS6	O25			
13	LT+	CS7	O114	L	L	L
14	ST+	CS14		L	L	L
15	ST+	CFA/I,CS21		L		L
16	LT+ST+	CS45+CS6			L	L
17	LT+	CS7				
18	LT+ST+	CS5+CS6				
19	LT+ST+	CFA/I, CS21		L	L	L
20	LT+ST+	CS1+CS3,CS21	O6	L	L	
21	LT+ST+	CS2+CS3	O6		L	
22	LT+ST+	NEG				
23	LT+ST+	NEG				
24	ST+	NEG				
25	LT+	NEG		L	L	
26	ST+	NEG				
27	LT+	NEG		L	L	L
28	ST+	NEG				
29	LT+ST+	NEG				
30	LT+	NEG			L	L
31	ST+	CFA/I	O126	L	L	
32	LT+ST+	CFA/I				
33	LT+ST+	CS1+CS3,CS21			L	
34	ST+	CS6				
35	LT+	NEG		L	L	L
36	EPEC					
37	EPEC					
38	EPEC					
39	EPEC				L	L
40	EPEC				L	L

Note: Of the 40 pathogenic *E. coli* isolates, 35 were identified as ETEC strains by the presence of the heat-stabile (ST) or heat-labile (LT) enterotoxin. The strains were further characterized by the presence of colonization factor (CF) antigens and by O serotype. Five strains were EPEC isolates. The lysis (L) as observed in the 10 ml test tube lysis assay on the indicated host strain is indicated for a 9-phage (Table 1) and 14-phage T4-like phage cocktail (NPC 1000, NPC 1001, NPC 1002, NPC 1003, NPC 1004, NPC 1005, NPC 1009, NPC 1032, NPC 1040, NPC 1041, NPC 1042, NPC 1043, NPC 1044, NPC 2017) and for the commercial Microgen phage cocktail. Underlined values from the 9-phage cocktail were confirmed by plaque assay. NEG, negative for tested CF.

### Coverage of *E. coli* pathogens from a community diarrhoea survey in Bangladesh

The commercial Russian phage cocktail was presumably selected for good coverage on *E. coli* strains from Russia. Its low coverage on the Mexican *E. coli* pathogens raises the question whether phage cocktails display geographical specificities. We do not know the selection process of phages for the Microgen cocktail, but at the Eliava Institute in Tbilisi/Georgia where Soviet phage therapy took its origin, new phages against *E. coli* pathotypes not covered by the previous stock are in regular intervals added to the cocktail (Chanishvili, 2012). To test the hypothesis of geographical diversity, we studied the coverage of the above-mentioned phage cocktails against 40 *E. coli* isolates from a community survey of childhood diarrhoea in Bangladesh (Qadri *et al*., 2007). In contrast to the Mexican collection, only 5 isolates represented EPEC strains while 35 strains were ETEC strains with different combinations of ST and LT enterotoxins and colonization factors as assessed by ELISA and monoclonal antibody-based dot blot assays, respectively (Table 2). Our nine-phage cocktail lysed only 30% of the pathogenic *E. coli* strains from Bangladesh compared with its 69% coverage on the Mexican strains. We then increased the number of constituting T4-like phages to 14 in the cocktail, based on the lytic properties of individual phage strains from our cocktail against ETEC strains from Bangladesh. We achieved 53% coverage, while the Microgen phage cocktail showed 33% coverage on this strain collection from Bangladesh compared with 19% coverage it had achieved on the Mexican collection. For a subset of the *E. coli* strains, the phage susceptibility of the strain was confirmed by the plaque assay (Table 2).

### Coverage of *E. coli* pathogens from diarrhoea patients hospitalized in Bangladesh

The distinct coverage observed with the same phage cocktail on different sets of *E. coli* pathogens could reflect the difference in location (Mexico *versus* Bangladesh) or pathotype (EPEC *versus* ETEC). To separate both effects, we tested the cocktails against different pathotypes of *E. coli* obtained from the same geographical region. From the Enteric Microbiology and Immunology Units of the International Center for Diarrheal Disease Research in Dhaka/Bangladesh (icddr,b) we obtained *E. coli* isolates that represented all major pathotypes, namely 15 EPEC, 15 ETEC, 10 enteroaggregative (EAEC), 3 enteroinvasive (EIEC), and 3 Verotoxin-producing (VTEC) *E. coli* strains, all isolated from diarrhoea patients hospitalized in 2008 at icddr,b. Our T4-like phage cocktail consisting of nine phages lysed 24% of these *E. coli* strains while the Microgen phage cocktail showed 50% coverage (Table 3). Two different batches of Microgen ColiProteus phage cocktails showed a nearly identical lysis pattern on these strains (data not shown) while two different batches from Microgen ‘Sextaphage’ cocktail showed an overlapping, but distinct lysis pattern with an overall lower coverage of 35% (Table 3). Since we were not satisfied with the 24% coverage by our nine-phage cocktail, we modified its composition to obtain a broader coverage. Modification was done on the basis of lytic host range determined for each individual phage strain against the *E. coli* pathogens isolated in 2008 from patients at icddr,b. A new cocktail consisting of 10 T4-like phages led to an increased coverage of 50% on these hospital-derived *E. coli* pathogens. With the 14-phage T4 cocktail, 61% coverage was achieved (Table 3).

**Table 3:** Lysis of *E. coli* strains representing different pathotypes isolated in 2008 from patients hospitalized with diarrhoea at icddr,b hospital in Dhaka, Bangladesh with the indicated phage cocktails

No	Pathotype	AGE	N 9-p	N 10-p	N 14-p	Micro	Sexta
1	EAggEC	P					
2	EAggEC	P			L	L	
3	EAggEC	P				L	
4	EAggEC	P	L	L	L	L	L
5	EAggEC	P			L		L
6	EAggEC		L	L	L	L	
7	EAggEC	P		L	L	L	L
8	EAggEC	P			L	L	
9	EAggEC	P		L	L	L	
10	EAggEC	P		L	L	L	L
11	EIEC		L	L	L	L	L
12	EIEC		L	L	L	L	L
13	EIEC		L	L	L	L	L
14	EPEC						
15	EPEC			L	L		
16	EPEC						
17	EPEC						
18	EPEC		L	L	L		
19	EPEC	P		L	L	L	L
20	EPEC	P					
21	EPEC	P					
22	EPEC	P	L	L	L		
23	EPEC	P				L	
24	EPEC					L	L
25	EPEC			L	L		
26	EPEC				L		L
27	EPEC	P					
28	EPEC	P		L	L		L
29	VTEC		L	L	L	L	L
30	VTEC		L	L	L	L	L
31	VTEC		L	L	L	L	
32	ETEC O114	P		L	L	L	
33	ETEC O167	P				L	L
34	ETEC O6	P			L		
35	ETEC O25	P		L	L		
36	ETEC O25	P					
37	ETEC O78	P		L	L	L	L
38	ETEC O115	P		L	L		
39	ETEC O114	P					
40	ETEC O115	P				L	L
41	ETEC O25	P				L	
42	ETEC O115	P					
43	ETEC O6	P	L	L	L		
44	ETEC O167	P					
45	ETEC O114	P		L	L	L	
46	ETEC O8	P					

Notes: Pathotypes: Enteroaggregative *E. coli* (EAggEC), Enteroinvasive *E. coli* (EIEC), Enteropathogenic *E. coli* (EPEC), Verotoxin producing *E. coli* (VTEC), Enterotoxigenic *E. coli* (ETEC) – the latter were serotyped for O antigen. Age: P indicates pediatric patient. Phage cocktails: N9-p cocktail consisting of 9 T4-like phages, N10-p cocktail consisting of 10 T4-like phages (for composition see Table 1); N14-p cocktail consisting of 14 T4-like phages (for composition see Table 2), Micro: ColiProteus phage cocktail; Sexta: Sextaphage cocktail from Microgen. L: lysis in 10 ml test tube assay.

Notably, the 10-phage cocktail lysed only 46% of the Mexican strains, less than the 68% lysed by the 9-phage cocktail (Table 1). Changing the phage composition of a cocktail in order to achieve a higher coverage in one geographical region might thus result in lesser coverage in another region. *E. coli* pathogen coverage by a phage cocktail is thus strongly context-dependent, suggesting that phages for therapy should be tested, if not isolated, using the most prevalent pathogenic strains in a given geographical area.

In 2011, we requested again random ETEC and EPEC isolates from children hospitalized with diarrhoea at icddr,b to explore temporal variation of pathogen coverage by given phage cocktails. The 10-phage T4 cocktail showed 29% coverage in the tube lysis test. The T4-like phage cocktail showed phage plaque counts in excess of 10^4^ pfu/ml in all cases where lysis was observed (Table 4). The Microgen Coliproteus phage cocktail lysed only 6% of these test strains, but formed plaques on half of them, but plaque counts were low when the cells were not lysed. Apparently, plaque counts higher than 2 × 10^3^ pfu/ml are needed to cause lysis of the cells (Table 4). Lysis was occasionally observed when no plaques were seen in the plaque assay. Whether this represents ‘lysis from without’ by phage tail-associated lytic enzymes from phages that cannot complete an infection cycle or ‘abortive infection’ is not clear, but according to the low moi which we used and a theoretical paper (Abedon, 2011), the second diagnosis is more appropriate. Independent of this question of clear terminology, bacterial cells do not experience the same environment when they are grown in solid medium as compared to liquid that leads to a distinct expression of some surface proteins which may affect the infection process.

**Table 4:** Phage susceptibility of ETEC and EPEC strains isolated from pediatric diarrhoea patients hospitalized in 2011 at icddr,b, Dhaka, Bangladesh as assessed by tube lysis and plaque assay

No	Pathotype	N10-p	N10-p	Micro	Micro
		lysis	pfu/ml	lysis	pfu/ml
1	ETEC		< 10		< 10
2	ETEC		< 10		< 10
3	EPEC		< 10	lysis	4 × 10^5^
4	ETEC		< 10		< 10
5	ETEC		< 10		< 10
6	EPEC		< 10		< 10
7	ETEC		< 10		< 10
8	EPEC		< 10	(lysis)	20
9	EPEC	lysis	1 × 10^5^		200
10	ETEC	lysis	1 × 10^5^		50
11	EPEC		< 10		2000
12	ETEC		< 10		< 10
13	ETEC	lysis	5 × 10^4^		10
14	ETEC		< 10		< 10
15	ETEC	lysis	1 × 10^5^		10
16	EPEC		< 10		300
17	ETEC	lysis	1 × 10^5^		2000

Since *Shigella* is taxonomically not even a subspecies of *E. coli*, we extended the testing to *Shigella* isolates from 10 different pediatric patients. The isolates represented two different *Shigella* ‘species’; one was represented with four different serotypes. All strains showed the *ipaH* virulence genes, while none contained the Shiga toxin gene. The Microgen ColiProteus phage cocktail formed plaques on 9 *Shigella* isolates while 10 T4-like phage cocktail showed lysis and plaques on 6 *Shigella* isolates (Table 5).

**Table 5:** Lysis of Shigella strains from patients at icddr,b, Dhaka, Bangladesh

Species	Virulence gene	Serotype	N10-p	Microgen
*Shigella flexneri*	ipaH	1b	< 10	1 × 10^4^
*S. flexneri*	ipaH	2a	< 10	1 × 10^4^
*S. flexneri*	ipaH	3a	< 10	2 × 10^4^
*S. flexneri*	ipaH	6	< 10	< 10
*S. flexneri*	ipaH	6	1 × 10^3^	2 × 10^5^
*Shigella sonnei*	ipaH		1 × 10^8^	3 × 10^6^
*S. sonnei*	ipaH		9 × 10^7^	2 × 10^6^
*S. sonnei*	ipaH		5 × 10^7^	5 × 10^4^
*S. sonnei*	ipaH		1 × 10^5^	1 × 10^5^
*S. sonnei*	ipaH		9 × 10^7^	3 × 10^6^

## Discussion

T4-like phages from our collection were with few exceptions all isolated on a non-pathogenic laboratory *E. coli* strain, more specifically K803, a derivative of the K-12 strain lacking the lambda prophage (Chibani-Chennoufi *et al*., 2004b). This restriction could be a significant selection bias which might explain the relatively poor coverage observed in the current study. One might object that phages should be isolated using prevalent pathogenic *E. coli* as indicator strains even if this renders the whole experimental work more difficult to handle (need of a safety laboratory, need of many propagating strains). If these phages are subsequently propagated on a non-pathogenic laboratory strain for commercial purposes, then their ability to lyse the most prevalent pathogenic strains should be confirmed. In fact, in the initial phase of our work, we isolated phages from stool samples of diarrhoea patients using in parallel an enteropathogenic *E. coli* strain, which was commonly isolated from childhood diarrhoea cases, and the laboratory K803 *E. coli* strain (Chibani-Chennoufi *et al*., 2004b). We obtained less phage isolates on the pathogenic strain than on the laboratory *E. coli* strain. Furthermore, the phage isolates made on the pathogenic *E. coli* strain were *Siphoviridae* with 50 kb genomes showing the morphology of Jersey- and beta-4 like phages and a narrower lytic pattern on O-serotyped pathogenic *E. coli* strains than phage isolates from the laboratory K803 strain, which yielded *Myoviridae* with a 170 kb genome, the morphology of T4-like phages and a broader host range on O-serotyped pathogenic *E. coli* strains (Chibani-Chennoufi *et al*., 2004b). We also obtained rather narrow host-range phage isolates when using a few other pathogenic *E. coli* as indicator strains (unpublished observations).

It is not unusual to select and propagate phages for phage therapy purpose against pathogenic bacteria on a nonpathogenic host strain. For example, a multivalent *Salmonella* phage was selected and amplified on a non-pathogenic *E. coli* strain. After serial passage on the two alternative hosts, the researchers observed a smaller burst size and longer rise period for the phage propagated on the heterologous *E. coli* host than on the homologous *Salmonella* host, but the lower cost of producing a safer phage product justified in their opinion these growth disadvantages. Notably, no difference in host range was observed between the phage propagated on the pathogenic *Salmonella* or non-pathogenic *E. coli* host (Santos *et al*., 2010).

Propagation on a laboratory strain avoids biosafety production conditions and the risk of transfer of bacterial or prophage-encoded toxins (Canchaya *et al*., 2003) from the pathogenic host into the phage preparation. The fact that K-12 displays only a truncated LPS molecule with a shortened O side chain (Qimron *et al*., 2006) might not only have changed biological phenotypes like pathogenicity for infection models in worms (Browning *et al*., 2013). Use of K-12 might have selected against phages recognizing the O side chain-specific sugar residues as receptor potentially explaining why the phages selected on the laboratory *E. coli* strain showed a broader host range on O-serotyped pathogenic *E. coli* than the phages selected on a pathogenic *E. coli* strain displaying a complete O side chain. While the majority of our phage isolates still showed a rather narrow infection range on our pathogenic *E. coli* strain collection, only few of them were actually O serotype-specific. We do not know what receptor structures our phages recognized. Some researchers had demonstrated that the conserved inner parts of the LPS molecule can serve as receptors for T4 and T7 (Tamaki *et al*., 1971; Qimron *et al*., 2006), potentially explaining the infection of many different O serotypes with a single phage. Recognition of alternative T4 phage receptors like the membrane protein OmpC proposed by other researchers (Hashemolhosseini *et al*., 1994; Yu *et al*., 2000) is not very likely for the isolated phages since one would expect a much broader cross-reactivity on O-serotypes *E. coli* pathogens than was actually observed.

One might also object that determining the host range of the isolated phages on *E. coli* growing in LB broth as done for enterobacteria propagation in the laboratory has nothing in common with the conditions encountered *in vivo*. For example, in our experiments, the infected *E. coli* cells were shaken to assure oxygen access while the lower parts of the gut display anaerobic conditions. To address this problem, we tested whether the investigated T4-like phages could infect *E. coli* under anaerobic *in vitro* growth conditions. This was the case: our T4-like phages yielded *in vitro* similar titers under aerobic and anaerobic growth (Weiss *et al*., 2009). Of course, there are many other conditions that differentiate *in vitro* from *in vivo* growth (Brüssow, 2013). For example, in their natural environment bacteria grow mostly in biofilms while broth culture represents growth in suspension. Indeed, T4 phage showed lytic infections on biofilms (Doolittle *et al*., 1995), but nutrient limitation in biofilms delayed the lysis process (Doolittle *et al*., 1996). One might therefore wonder whether *in vitro* conditions better adapted to *in vivo* growth conditions would not be preferable to standard broth culture. Since the *in vivo* growth conditions of *E. coli* are still poorly characterized, it is unfortunately not yet clear what *in vitro* conditions best mimic the *in vivo* condition.

It is widely believed that phage therapy approaches need phage cocktails (Brüssow, 2012). If resistance development is an issue during phage therapy (Cairns *et al*., 2009), it is less likely to develop against a phage cocktail than against a single phage. However, combining phages into a cocktail can create interference problems making the actual lytic capacity of a phage cocktail less than the sum of its parts. This phenomenon was here observed with cocktails of T4-like phages. Mechanistically, super-infection of *E. coli* cells with two T4-like phages causes lysis inhibition (Abedon, 1992) preventing an OD decrease, which was the read-out of our test. We tried different combinations of individual T4-like phages hoping to identify specific phage strain combinations displaying antagonistic behavior in order to eliminate them from the cocktail. However, no such specific interference problems between individual phage strains were spotted, suggesting merely random effects when increasing the overall number of T4-like phages in a cocktail. Retrospectively, one might also ask whether limiting the phage cocktail to a single phage genus like T4-like phages for safety reasons was a good strategy. Safety trials showed no difference when using a T4-like phage cocktail (Sarker *et al*., 2012) or the Russian phage cocktail containing in addition to T4- and T7-like phages a dozen further phage genera (McCallin *et al*., 2013). As the *in vivo* phage growth conditions in the human gut are still largely undefined, working with a complex phage cocktail as done by the Eastern European phage tradition might represent a hedge betting strategy for diversifying risk. However, when considering only the coverage of the target pathogen with *in vitro* lysis tests, no clear superiority of the Microgen phage cocktails over the T4-like phage cocktail was observed.

If we accept the hypothesis that *in vitro* lysis is a necessary, although not sufficient condition for *in vivo* lysis, coverage is critical for efficient phage therapy. To illustrate it numerically: with 50% coverage, two *E. coli* patients need to be treated to expect an effect in one of them. If only one out of two to three hospitalized pediatric diarrhoea patients suffers from an *E. coli* diarrhoea as is the case in Bangladesh (Albert *et al*., 1999), four to six patients need to be treated for one to profit from phage treatment. This calculation neglects the possibility that many patients are infected with similar strains which is actually the case during a given epidemic (Qadri *et al*., 2005), while this might not be the same strains between epidemics (Chowdhury *et al*., 2011). This consideration put aside, relatively large numbers of children with diarrhoea need to be enrolled into a controlled clinical trial to observe a treatment effect raising even an ethical conundrum using a therapeutical preparation which only cures a limited proportion of the patients. This problem could be solved when targeting *Shigella* infections. Dysentery symptoms are so characteristic that it can be easily diagnosed clinically and its coverage by phage was much higher than for *E. coli* diarrhoea. The combination of both factors makes it likely that at least every second dysentery patient could profit from phage therapy. In line with other reports (Chang and Kim, 2011), *Shigella* was lysed by coliphages, but this should not be surprising since *Shigella* qualifies not even as a subspecies of *E. coli* (Sims and Kim, 2011). In support to this conclusion identifying *Shigella* infections as a suitable target for phage therapy, the only clinical trial published by Soviet phage therapy researcher reported successful prevention of *Shigella* dysentery (Sulakvelidze *et al*., 2001). Diarrhoea-associated *E. coli* pathogens in contrast are a difficult target for phage therapy since the pathogen coverage by phages varies with geographical region, epidemiological setting and time as demonstrated in the present study. It is not clear whether our observations can be generalized. Due to frequent inundation by flood water, Bangladesh displays an exceptionally high dynamic of diarrhoea pathogens (Harris *et al*., 2008) making it not a typical, but a worst-case scenario.

Evolutionary biologists have asked whether *in vitro* phage-host systems are adequate models for the *in situ* situation in the real ecological niche (Forde *et al*., 2008). For clinical efficacy, phages must display the appropriate *in vivo* stability to survive the gastro-intestinal passage, a good *in situ* bioavailability at their site of action and a good replication potential on *E. coli* demonstrating different physiological states in different gut segments. None of these phenotypes can be easily tested *in vitro*. Some pathogenic *E. coli* strains multiply in the small intestine (e.g. EPEC), others replicate in the large intestine (e.g. EHEC, *Shigella*). Data on intestinal phage–host interaction in laboratory mice were equivocal. In our laboratory, a T4-like phage was less efficient than a T7-like phage in replicating on slowly growing commensal *E. coli* in the gut (Weiss *et al*., 2009). In another laboratory, a T4-like phage was a better replicator than a T7-like phage on a pathogenic *E. coli* strain asymptomatically colonizing the gut of mice (Maura *et al*., 2012a,b). In both models, oral T7-like phages replicated to high titers in the gut without, however, having a sustained effect on the fecal *E. coli* titer (Weiss *et al*., 2009; Maura *et al*., 2012a). When investigating phage–host interaction in different segments of the gut, marked differences were observed (Weiss *et al*., 2009; Maura *et al*., 2012b). Our understanding of coliphage *E. coli* interaction in the gut, their natural ecological niche, is apparently so limited (Brüssow, 2013) that testing a phage cocktail for *in vitro* coverage can only be seen as a step towards a successful phage therapy approach. In the absence of an *E. coli* diarrhoea model in small laboratory animals, we study currently the faecal excretion pattern in children hospitalized with *E. coli* diarrhoea who were treated with two different phage cocktails to get data on the *in vivo* replication of oral phage cocktails.

## References

[b1] Abedon ST (1992). Lysis of lysis-inhibited bacteriophage T4-infected cells. J Bacteriol.

[b2] Abedon ST (2011). Lysis from without. Bacteriophage.

[b3] Albert MJ, Faruque SM, Faruque ASG, Neogi PKB, Ansaruzzaman M, Bhuiyan NA (1995). Controlled study of *Escherichia coli* diarrheal infections in Bangladeshi children. J Clin Microbiol.

[b4] Albert MJ, Faruque AS, Faruque SM, Sack RB, Mahalanabis D (1999). Case-control study of enteropathogens associated with childhood diarrhea in Dhaka, Bangladesh. J Clin Microbiol.

[b5] Browning DF, Wells TJ, França FL, Morris FC, Sevastsyanovich YR, Bryant JA (2013). Laboratory adapted *Escherichia coli* K-12 becomes a pathogen of *Caenorhabditis elegans* upon restoration of O antigen biosynthesis. Mol Microbiol.

[b6] Bruttin A, Brüssow H (2005). Human volunteers receiving *Escherichia coli* phage T4 orally: a safety test of phage therapy. Antimicrob Agents Chemother.

[b7] Brüssow H (2012). What is needed for phage therapy to become a reality in Western medicine?. Virology.

[b8] Brüssow H (2013). Bacteriophage-host interaction: from splendid isolation into a messy reality. Curr Opin Microbiol.

[b9] Cairns BJ, Timms AR, Jansen VA, Connerton IF, Payne RJ (2009). Quantitative models of in vitro bacteriophage-host dynamics and their application to phage therapy. PLoS Pathog.

[b10] Canchaya C, Proux C, Fournous G, Bruttin A, Brüssow H (2003). Prophage genomics. Microbiol Mol Biol Rev.

[b11] Chang HW, Kim KH (2011). Comparative genomic analysis of bacteriophage EP23 infecting *Shigella sonnei* and *Escherichia coli*. J Microbiol.

[b12] Chanishvili N (2012). Phage therapy-history from Twort and d'Herelle through Soviet experience to current approaches. Adv Virus Res.

[b13] Chibani-Chennoufi S, Sidoti J, Bruttin A, Kutter E, Sarker S, Brüssow H (2004a). In vitro and in vivo bacteriolytic activities of *Escherichia coli* phages: implications for phage therapy. Antimicrob Agents Chemother.

[b14] Chibani-Chennoufi S, Sidoti J, Bruttin A, Dillmann ML, Kutter E, Qadri F (2004b). Isolation of *Escherichia coli* bacteriophages from the stool of pediatric diarrhea patients in Bangladesh. J Bacteriol.

[b15] Chowdhury F, Rahman MA, Begum YA, Khan AI, Faruque AS, Saha NC (2011). Impact of rapid urbanization on the rates of infection by *Vibrio cholerae* O1 and enterotoxigenic *Escherichia coli* in Dhaka, Bangladesh. PLoS Negl Trop Dis.

[b16] Cravioto A, Reyes R, Trujillo F, Uribe F, Navarro A, De la Roca J (1990). Risk of diarrhea during the first year of life associated with initial and subsequent colonization by specific enteropathogens. Am J Epidemiol.

[b17] Doolittle MM, Cooney JJ, Caldwell DE (1995). Lytic infection of *Escherichia coli* biofilms by bacteriophage T4. Can J Microbiol.

[b18] Doolittle MM, Cooney JJ, Caldwell DE (1996). Tracing the interaction of bacteriophage with bacterial biofilms using fluorescent and chromogenic probes. J Ind Microbiol.

[b19] Flores CO, Meyer JR, Valverde S, Farr L, Weitz JS (2011). Statistical structure of host-phage interactions. Proc Natl Acad Sci U S A.

[b20] Forde SE, Beardmore RE, Gudelj I, Arkin SS, Thompson JN, Hurst LD (2008). Understanding the limits to generalizability of experimental evolutionary models. Nature.

[b21] Gunzburg ST, Tornieporth NG, Riley LW (1995). Identification of enteropathogenic *Escherichia coli* by PCR-based detection of the bundle-forming pilus gene. J Clin Microbiol.

[b22] Harris AM, Chowdhury F, Begum YA, Khan AI, Faruque AS, Svennerholm AM (2008). Shifting prevalence of major diarrheal pathogens in patients seeking hospital care during floods in 1998, 2004, and 2007 in Dhaka, Bangladesh. Am J Trop Med Hyg.

[b23] Hashemolhosseini S, Montag D, Krämer L, Henning U (1994). Determinants of receptor specificity of coliphages of the T4 family. A chaperone alters the host range. J Mol Biol.

[b24] Heinrichs DE, Yethon JA, Whitfield C (1998). Molecular basis for structural diversity in the core regions of the lipopolysaccharides of *Escherichia coli* and *Salmonella enterica*. Mol Microbiol.

[b25] Karch H, Böhm H, Schmidt H, Gunzer F, Aleksic S, Heesemann J (1993). Clonal structure and pathogenicity of Shiga-like toxin-producing, sorbitol-fermenting *Escherichia coli* O157:H. J Clin Microbiol.

[b26] Kelly D, McAuliffe O, Ross RP, O'Mahony J, Coffey A (2011). Development of a broad-host-range phage cocktail for biocontrol. Bioeng Bugs.

[b27] McCallin SE, Sarker SA, Barretto C, Sultana S, Berger B, Huq S (2013). Safety analysis of a Russian phage cocktail: from metagenomic analysis to oral application in healthy human subjects. Virology.

[b28] McConnell MM, Chart H, Field H, Hibberd M, Rowe B (1989). Characterization of a putative colonization factor (PCFO166) of enterotoxigenic *Escherichia coli* of serogroup O166. J Gen Microbiol.

[b29] Maura D, Morello E, du Merle L, Bomme P, Le Bouguénec C, Debarbieux L (2012a). Intestinal colonization by enteroaggregative *Escherichia coli* supports long-term bacteriophage replication in mice. Environ Microbiol.

[b30] Maura D, Galtier M, Le Bouguénec C, Debarbieux L (2012b). Virulent bacteriophages can target O104:H4 enteroaggregative *Escherichia coli* in the mouse intestine. Antimicrob Agents Chemother.

[b31] Merril CR, Biswas B, Carlton R, Jensen NC, Creed GJ, Zullo S, Adhya S (1996). Long-circulating bacteriophage as antibacterial agents. Proc Natl Acad Sci U S A.

[b32] Ørskov F, Ørskov I (1984). Serotyping of *Escherichia coli*. Meth Microbiol.

[b33] Payne RJ, Phil D, Jansen VA (2000). Phage therapy: the peculiar kinetics of self-replicating pharmaceuticals. Clin Pharmacol Ther.

[b34] Pollard DR, Johnson WM, Lior H, Tyler SD, Rozee KR (1990). Rapid and specific detection of verotoxin genes in *Escherichia coli* by the polymerase chain reaction. J Clin Microbiol.

[b35] Qadri F, Das SK, Faruque ASG, Fuchs GJ, Albert MJ, Sack RB, Svennerholm A-M (2000). Prevalence of toxin types and colonization factors in enterotoxigenic *Escherichia coli* isolated during a 2-year period from diarrheal patients in Bangladesh. J Clin Microbiol.

[b36] Qadri F, Khan AI, Faruque AS, Begum YA, Chowdhury F, Nair GB (2005). Enterotoxigenic *Escherichia coli* and *Vibrio cholerae* diarrhea, Bangladesh, 2004. Emerg Infect Dis.

[b37] Qadri F, Saha A, Ahmed T, Al Tarique A, Begum YA, Svennerholm AM (2007). Disease burden due to enterotoxigenic *Escherichia coli* in the first 2 years of life in an urban community in Bangladesh. Infect Immun.

[b38] Qimron U, Marintcheva B, Tabor S, Richardson CC (2006). Genomewide screens for *Escherichia coli* genes affecting growth of T7 bacteriophage. Proc Natl Acad Sci U S A.

[b39] Robins-Browne RM (1987). Traditional enteropathogenic *Escherichia coli* of infantile diarrhea. Rev Infect Dis.

[b40] Santos SB, Fernandes E, Carvalho CM, Sillankorva S, Krylov VN, Pleteneva EA (2010). Selection and characterization of a multivalent Salmonella phage and its production in a nonpathogenic *Escherichia coli* strain. Appl Environ Microbiol.

[b41] Sarker SA, McCallin S, Barretto C, Berger B, Pittet AC, Sultana S (2012). Oral T4-like phage cocktail application to healthy adult volunteers from Bangladesh. Virology.

[b42] Schultsz C, Pool GJ, van Ketel R, de Wever B, Speelman P, Dankert J (1994). Detection of enterotoxigenic *Escherichia coli* in stool samples by using nonradioactively labeled oligonucleotide DNA probes and PCR. J Clin Microbiol.

[b43] Schwarzer D, Buettner FF, Browning C, Nazarov S, Rabsch W, Bethe A (2012). A multivalent adsorption apparatus explains the broad host range of phage phi92: a comprehensive genomic and structural analysis. J Virol.

[b44] Sims GE, Kim SH (2011). Whole-genome phylogeny of *Escherichia coli/Shigella* group by feature frequency profiles (FFPs). Proc Natl Acad Sci U S A.

[b45] Sulakvelidze A, Alavidze Z, Morris JG (2001). Bacteriophage therapy. Antimicrob Agents Chemother.

[b46] Svennerholm A-M, Wiklund G (1983). Rapid GM1-enzyme-linked immunosorbent assay with visual reading for identification of *Escherichia coli* heat-labile enterotoxin. J Clin Microbiol.

[b47] Svennerholm AM, Wikström M, Lindblad M, Holmgren J (1986). Monoclonal antibodies against *Escherichia coli* heat-stable toxin (STa) and their use in a diagnostic ST ganglioside GM1-enzyme-linked immunosorbent assay. J Clin Microbiol.

[b48] Tamaki S, Sato T, Matsuhashi M (1971). Role of lipopolysaccharides in antibiotic resistance and bacteriophage adsorption of *Escherichia coli* K-12. J Bacteriol.

[b49] Thomassen E, Gielen G, Schütz M, Schoehn G, Abrahams JP, Miller S, van Raaij MJ (2003). The structure of the receptor-binding domain of the bacteriophage T4 short tail fibre reveals a knitted trimeric metal-binding fold. J Mol Biol.

[b50] Weiss M, Denou E, Bruttin A, Serra-Moreno R, Dillmann ML, Brüssow H (2009). In vivo replication of T4 and T7 bacteriophages in germ-free mice colonized with *Escherichia coli*. Virology.

[b51] Weitz JS, Poisot T, Meyer JR, Flores CO, Valverde S, Sullivan MB, Hochberg ME (2013). Phage-bacteria infection networks. Trends Microbiol.

[b52] Yu SL, Ko KL, Chen CS, Chang YC, Syu WJ (2000). Characterization of the distal tail fiber locus and determination of the receptor for phage AR1, which specifically infects *Escherichia coli* O157:H7. J Bacteriol.

